# VOICE–Validating Outcomes by Including Consumer Experience: A Study Protocol to Develop a Patient Reported Experience Measure for Aboriginal and Torres Strait Islander Peoples Accessing Primary Health Care

**DOI:** 10.3390/ijerph20010357

**Published:** 2022-12-26

**Authors:** Amal Chakraborty, Emma Walke, Roxanne Bainbridge, Ross Bailie, Veronica Matthews, Sarah Larkins, Paul Burgess, Deborah Askew, Erika Langham, Samantha Smorgon, Girish Swaminathan, Danielle Cameron, Tracey Piccoli, Megan Passey

**Affiliations:** 1 University Centre for Rural Health, The University of Sydney, Lismore, NSW 2480, Australia; 2 Poche Centre for Indigenous Health, Faculty of Health and Behavioural Sciences, The University of Queensland, St Lucia, QLD 4067, Australia; 3 College of Medicine and Dentistry, James Cook University, Townsville, QLD 4811, Australia; 4 NT Health, Northern Territory Government, Casuarina, NT 0811, Australia; 5 School of Medicine, Faculty of Medicine, The University of Queensland, St Lucia, QLD 4067, Australia; 6 School of Public Health, Faculty of Medicine, The University of Queensland, St Lucia, QLD 4067, Australia; 7 The Royal Australian College of General Practitioners (RACGP), East Melbourne, VIC 3002, Australia; 8 Australian Commission on Safety and Quality in Health Care (ACSQHC), Sydney, NSW 2000, Australia

**Keywords:** primary health care, patient reported experience measures, accreditation, aboriginal, feedback, participatory research, health systems strengthening, continuous quality improvement, community engagement, patient-centred care

## Abstract

Aboriginal and Torres Strait Islander peoples’ (hereafter respectfully referred to as Indigenous Australians) experiences of health care are shaped by historical, social and cultural factors, with cultural security critical to effective care provision and engagement between services and community. Positive patient experiences are associated with better health outcomes. Consequently, it is an accreditation requirement that primary health care (PHC) services must formally gather and respond to patient feedback. However, currently available patient feedback tools were not developed with Indigenous Australians, and do not reflect their values and world views. Existing tools do not capture important experiences of care of Indigenous Australians in PHC settings, nor return information that assists services to improve care. Consistent with the principles of Indigenous Data Sovereignty, we will co-design and validate an Indigenous-specific Patient Reported Experience Measure (PREM) that produces data by and for community, suitable for use in quality improvement in comprehensive PHC services. This paper presents the protocol of the study, outlining the rationale, methodologies and associated activities that are being applied in developing the PREM. Briefly, guided by an Aboriginal and Torres Strait Islander Advisory Group, our team of Indigenous and non-Indigenous researchers, service providers and policy makers will use a combination of Indigenous methodologies, participatory, and traditional western techniques for scale development. We will engage PHC service staff and communities in eight selected sites across remote, regional, and metropolitan communities in Australia for iterative cycles of data collection and feedback throughout the research process. Yarning Circles with community members will identify core concepts to develop an “Experience of Care Framework”, which will be used to develop items for the PREM. Staff members will be interviewed regarding desirable characteristics and feasibility considerations for the PREM. The PREM will undergo cognitive and psychometric testing.

## 1. Introduction

Comprehensive primary health care (PHC) is the most important part of the health system and is vital to improving health outcomes and reducing health inequities [1]. One strategy to improve the PHC system is to enhance processes for patient input to drive the system towards a more patient-centred model of care. The importance of patient-centred care in improving health care quality is widely recognised.

Experiences of health care are shaped by historical, social and cultural factors [2,3]. Culturally secure and positive care experiences are critical for improving health outcomes of Aboriginal and Torres Strait Islander peoples (hereafter respectfully referred to as Indigenous Australians), through greater engagement with care provision and self-management [3,4]. There is strong evidence that better patient experiences of care are associated with improved engagement and health outcomes including self-rated and objectively measured health outcomes, adherence to recommended treatment, greater uptake of preventive care, efficient use of health care resources, higher technical quality of care, and fewer adverse events [4]. When coupled with continuous quality improvement (CQI) processes, Patient Reported Experience Measures (PREMs) data can enable services to identify and thus act on areas in need of improvement and monitor changes over time. However, for these benefits to be realized, the data collected must reflect the values of the relevant community members as health service users (hereafter referred to as consumers); and must provide ‘actionable’ information to support quality improvement.

Given the strong evidence that positive patient experiences are associated with better health outcomes, routine collection and use of PREMs is a policy goal and an accreditation requirement within the Royal Australian College of General Practices’ (RACGP) Standards for general practices (https://www.racgp.org.au/running-a-practice/practice-standards) (website access date 9 October 2022) and under the Australian Commission on Safety and Quality in Health Care’s (ACSQHC) various accreditation schemes [5,6]. 

For Indigenous Australians, good health is not just a matter of having access to bio medical services and medications, or the absence of disease, but holistic wellbeing of the whole community and the environment, essential for strong kinship and culture [7]. In other words, as Garvey et al. [8] highlighted, Indigenous peoples’ “collectivist worldview” respects the well-being of the whole group over individual needs, which is significantly different from the dominant Western “individual-centric worldview”. However, existing tools do not capture this complexity nor support care quality improvement in Indigenous PHC settings as they were not developed or validated with Indigenous Australians, and do not reflect their values, beliefs and world views. Consequently, they do not adequately capture important experiences of care of Indigenous Australians, or return information that is meaningful for improving health service delivery. With strong Indigenous leadership, a participatory research project entitled ‘Validating Outcomes by Including Consumer Experience (VOICE)’ addresses this vital gap in currently available resources.

The VOICE Project was conceived through discussions with PHC services and policy partners and initiated at their request. Concerns with existing tools expressed by our partners, include culturally inappropriate language, failure to capture important concepts, a focus on an individual clinician rather than comprehensive PHC teams, failure to return ‘actionable’ information, and inability to support tracking of improvement over time or benchmarking. Critically, the current RACGP PREM, as commonly used by PHC services to address accreditation requirements, contains no questions that address cultural security, and no questions related to experiences of racism or discrimination. Yet, these incidents are commonly experienced by Indigenous Australians, including in health care settings [9]. Culturally unsafe practices, and experiences of racism are associated with disengagement from health services, poor chronic disease care, and poorer mental and physical health [2,10]. Self-determination and control is therefore critical to Indigenous peoples’ holistic health care and wellbeing [2,11], yet without mechanisms for input to service design and delivery that adequately capture their experiences, this is not feasible [12]. It is therefore imperative to develop a PREM that validly measures and captures relevant experiences of Indigenous primary health care consumers.

## 2. Materials and Methods

### 2.1. Study Aims

The VOICE project will co-design and validate an Indigenous-specific PREM for use in comprehensive PHC services. It will reflect the values and worldviews of Indigenous Australians, and be consistent with the principles of Indigenous Data Sovereignty [13,14]. Critically, the PREM will be developed from the ground up–not as an adaptation of existing tools. This ensures that Indigenous Australians exercise control of the creation of the tool and the data it generates, ensuring its relevance and that it respects individual and collective interests. The new PREM will enable PHC services to assess consumer perceptions of different service delivery models, and thus improve the quality of care they provide, enhance consumer engagement, improve health outcomes and reduce inequities in health and wellbeing experienced by Indigenous Australians. The study goal, aims and design were developed in partnership with participating services and other stakeholders. Our specific aims are to:

1. Identify elements of quality and experience of care that are most valued by Indigenous Australians attending PHC services to develop an Experience of Care Framework.
2. Engage with service providers and end-users (i.e., accreditation bodies, policymakers) to determine stakeholder views on the PREM, including how to ensure it: i) optimises feasibility of collection, analysis and interpretation of findings; and ii) can be adequately resourced for implementation.
3. Develop a PREM that captures valued experiences of care of Indigenous Australians in PHC settings; assess its face and content validity to ensure it covers elements that stakeholders consider most important; and ensure acceptability to consumers, services and other stakeholders.
4. Validate the PREM by assessing its psychometric properties and develop a suitable scoring system for the items that will be used for benchmarking and assessment of trends.

### 2.2. Context

The VOICE research project builds on ongoing work of a 20-year partnership of PHC providers, policy makers and researchers in Australia, all working to strengthen Indigenous PHC through collaborative research and implementation [15]. This research project is being undertaken as a key project for the Centre for Research Excellence: STRengthening systems for InDigenous health care Equity (CRE-STRIDE) (https://cre-stride.org/). The CRE-STRIDE is a national collaboration of researchers, service providers and policy makers aiming to improve the health and wellbeing of Indigenous Australians by improving the quality of the PHC they receive. CRE-STRIDE identified ‘Strengthening community input into improving quality improvement processes’ as one of four critical work streams. Within this stream, the VOICE project was established as a priority [16] in response to concerns repeatedly expressed by service partners regarding the inappropriateness of existing tools addressing Indigenous consumer experiences, as well as the importance of building mechanisms for community to drive health care improvement [17]. Our partners include national bodies involved in accreditation, the RACGP and the ACSQHC, state and territory level Indigenous Community-Controlled health organisations, the Northern Territory Government, two Primary Health Networks (PHNs) and Indigenous PHC services. 

In the Australian context, an Indigenous Community-Controlled organisation is a primary health care service initiated and operated by the local Indigenous community for holistic, comprehensive, and culturally appropriate health care delivery to the community, which is governed through a locally elected Board of Management [18]. While PHNs are independent organisations that coordinate primary health care services in their respective regions [19]. 

### 2.3. Overview of Research Approach and Methods

This study will use a systems-based participatory action research design to develop a PREM grounded in Indigenous peoples’ experience of comprehensive PHC. The PREM will be co-designed through an ‘All teach, all learn’ approach with community and stakeholders, thereby enhancing engagement across the system and rapid translation into policy and practice. The intent of the PREM is to facilitate systematic data driven CQI processes within PHC services based on consumer feedback [15,20].

The study is respectful of different cultural worldviews, values and practices, and draws on research approaches from both Indigenous and Western knowledge systems [7,21]. An Aboriginal and Torres Strait Islander Advisory Group (here in the Advisory Group) comprising Indigenous Australian representatives from participating communities and all Indigenous investigators of the VOICE project; and a Management Group composed of all Chief Investigators and Associate Investigators will oversee all phases of the study. The Advisory Group members will be identified and nominated by the VOICE Project’s partner PHC services and invited to participate in the project. These two groups will work together to provide advice and guidance to this project, with the Management Group providing governance oversight and the Indigenous Advisory Group providing expert advice on cultural integrity and ensuring the project remains connected to community concerns. 

**Figure 1:**
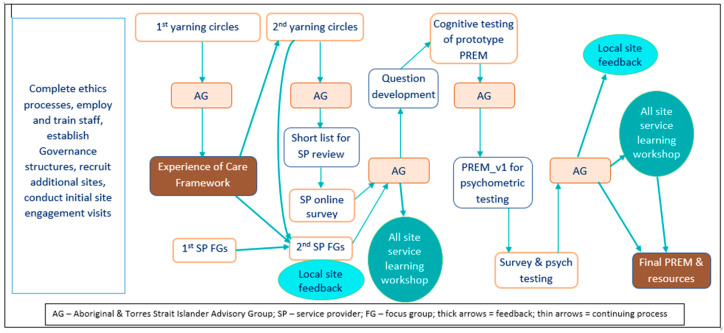
Study processes and overall activities.

The research process also has a strong knowledge translation (KT) orientation [22,23,24,25]. KT is integrated into VOICE Project implementation through the deliberate engagement of knowledge users (e.g., consumers, clinicians, health services, policymakers) from the outset as active participants in all stages of the research, from needs identification to dissemination and use of the PREM to improve systems of care. This participatory research approach has a strong social justice and self-determination orientation and recognises health care consumers as experts on the issues that impact their health and well-being [26]. To maximise benefit to communities and services throughout the research project, local site feedback mechanisms will be established to share and consider findings at several points throughout the project. The learnings from this process will be further enhanced through two combined ‘service learning’ workshops where services will consider commonalities and differences across the sites. Figure 1 illustrates the processes and overall activities of the VOICE study. 

### 2.4. Settings and Population

The VOICE research project will be conducted in eight sites across the Australian states and territories of New South Wales (NSW), Queensland (QLD), South Australia (SA) and the Northern Territory (NT) in remote, regional, and metropolitan settings. Sites include community controlled and government managed PHC services that primarily provide care to Indigenous people. 

#### 2.4.1. Initial Site Visits

Each of the services will initially be visited by the research team prior to any data collection to optimise engagement with the community and the service. Meetings will be held with key service staff, the service Board (or other advisory group depending on the service) and with other local elders and community groups consistent with local protocols and cultural norms. The main purposes of these meetings will be to ensure strong local involvement and support for the project at both the community and service level; and for the research team to learn about the local community, the service, meet key stakeholders and listen to communities’ issues relevant to the project.

#### 2.4.2. Ethical Review and Approval

The VOICE project followed the multi-site ethics application process with the Primary Committee being the Human Research Ethics Committee of NT Health and Menzies School of Health Research (NT HREC). In addition, we also sought ethical review and approval of this project from four other site relevant HRECs in NSW, QLD, SA and NT. The full list of reviewing HRECs and their respective project approval reference numbers are listed at the end of this article in the Institutional Review Board Statement section. 

### 2.5. Study Aims and Activities to Address Them

The methods to be used (see Figure 1) to achieve the aims of the project are described below. There will be an iterative cyclical process of data collection, analysis and interpretation where Aims 1 and 2 will be addressed simultaneously. 

#### 2.5.1. Aim 1: Identify Elements of Quality and Experience of Care That Are Most Valued by Aboriginal and Torres Strait Islander People Attending PHC Services

Data collection and analysis will progress in parallel and be underpinned by grounded theory methodology [27]. The approach enables development of a robust map of domains and their inter-relationships to develop an Experience of Care Framework. The framework will be used to identify core domains to be included in item development for the VOICE PREM.

##### Sampling, Participant Recruitment and Data Collection

Adult community members will be invited to collaborate in ‘story-telling’ activities (either in a ‘Yarning Circle’ (YC), or individually if preferred) unpacking participant-valued care and experiences. YCs are a group process that have often been applied in community settings in recent years as a way to safely engage with Indigenous participants to explore particular research questions [28]. In this context ‘Yarning’ provides a culturally sensitive way of representing the ‘diversities of truth’, whereby story-tellers maintain control [29]. ‘Yarning’ is a well-accepted Indigenous methodological approach to qualitative data collection because it facilitates in-depth discussions and allows participants to share their stories from the position of their lived experiences in an informal relaxed manner where they can talk freely about their experiences [30,31] and establish relationships with the researchers [32]. While this approach may have similarities with Focus Groups, ‘Yarning’ is less structured and focuses on privileging Indigenous voices to support improving health outcomes for Indigenous peoples and their communities giving participants greater control over the direction of the discussion [30].

Yarning does not follow the formal conventions of interviewing technique, rather it allows participants to ‘weave in and out’ of their stories while researchers listen for cues related to the research topic–in this case, patient experience of health care [28]. We will start with a purposive sample of 60–80 community members across genders, ages, service utilisation (frequently vs. rarely use the local service) and the eight sites (8–10 in each). Thereafter, theoretical sampling will be used to streamline data collection until saturation of data and categories [33] is reached. Participants will be recruited by our service partners and other community organisations (e.g., sports clubs) and receive a $50 gift card and a VOICE shirt to acknowledge their contributions and expertise. 

Two-three researchers, led by an Indigenous researcher, will convene and facilitate the YCs in collaboration with the local service. It is important to note here that the local service will assist with recruitment of participants and organising groups but not participate or be present at the YCs. This is to ensure community participants can speak freely without any fear or pressure. If a community participant prefers, they can also participate in a one-on-one yarning with a VOICE staff member to share their healthcare experiences and provide feedback with privacy. The YCs will involve both informal and formal Yarning and will take about 2–3 h. Formal Yarning and data collection will be preceded by a shared meal and informal yarning to develop trust, build relationship, maximise ease and ensure that participants are comfortable to share their stories in a group. This informal yarning will enable the research team and community participants to know each other better, with reciprocal sharing and learning from each other at the outset before data collection starts. Once the invited participants are settled after informal yarning, the lead facilitator will commence the formal Yarning with an open-ended question, “*Tell us your stories about your health care experience-what is important to you about your healthcare?*”. Other research team members accompanying the lead Indigenous facilitator will act as observers and/or assist with running the session (e.g., explaining the information sheet one-on-one to participants, signing of consent forms, recording sessions). The research team will also involve one male researcher to facilitate men’s YC groups if necessary. 

At each site, we will work with the local service and the community representative on the VOICE project Advisory Group to determine the most appropriate process for engaging community and recruiting participants. In addition to an Advisory Group representative, the partner health service will be asked to identify a local site champion/cultural mentor to support the process. The site champion will be trained by the research team regarding the project, including ethical issues, recruitment, and data collection, as required. 

A brief (4-point) guide for the community YCs has been developed to ensure key issues are covered, in an open yarning style. Participants will be encouraged to freely talk about a range of topics related to health, healthcare and how it relates to them as individuals and or their community. At the same time, we will ensure flexibility and depth in the discussion to aim for all participants’ perspectives to be explored. Participants will also be asked to complete a brief questionnaire to collect demographic data including age, gender, which Indigenous Country they identify with and frequency of utilisation of the health service. All participants will provide written consent. 

While details will vary with each site, it is anticipated that the study invitation will be shared using a combination of posters, information sheets, word-of-mouth and engagement through local social media (e.g., Facebook page for the local service or for the local community). We will be advised by our local partners regarding composition of YCs (e.g., mix of ages, gender, language groups, etc.). There is the option of running one or multiple YCs at each site to address cultural needs such as diversity of language groups, gender and to maximise comfort of participants.

##### Analysis

YCs will be audio-recorded and transcribed. Immediately following each YC the researchers will debrief and write memos. Transcripts, memos, and field notes will be coded, in an ‘all is data’ approach. A core group of Indigenous and non-Indigenous research team members with a range of relevant experience and expertise will conduct the analysis and interpretation of the data. An inductive analytical approach will be used to identify relevant codes and categories, with the use of memos to capture important insights. Following Sbaraini et al. [33,34], coding will be undertaken in three sequential stages in consultation with the VOICE project Advisory Group. First, in initial coding, the research team will generate as many ideas as possible from reviewing the data. Second, in focused coding, the research team will consolidate the initial codes into a selected set of central codes from the entire dataset. In this stage the research team will discuss and prioritise which initial codes are most prevalent or important and contribute most to the data analysis and synthesis. Third, in theoretical coding, the research team will refine the final categories and relate them to one another. Constant comparative methods, by comparing codes against codes and data against data, will enable exploration of issues to establish points of consensus and dissent and to saturate categories. 

##### Generating Domains and Factors for Consideration in the PREM 

The data collected from YCs will provide evidence of patient preferences, experiences and quality of care and include futurist elements of how care could be transformed to meet their needs. Grounded theory will be used to develop a robust conceptual theory or map of domains and their inter-relationships, to underpin item (question) development for use in the PREM [35,36]. This approach allows development of items reflecting the values and priorities of participants, captures Indigenous people’s voices, and makes transparent the process of domain development and item generation from verbatim concepts [35]. A causal-consequence model will be developed to identify the most important and meaningful domains in healthcare-what works for whom, in what contexts, under what conditions, through which mechanisms and with what consequences. 

Findings will be discussed iteratively with the research team and the Advisory Group to generate a conceptual framework (the Experience of Care Framework) once theoretical saturation is achieved. The framework, domains, factors, and their naming, will be reviewed by the Advisory Group to assess face and content validity, with sensitivity to language and constructs. Changes will be made prior to a concept prioritisation process. 

#### 2.5.2. Aim 2: Engage with Service Providers and End-Users (i.e., Accreditation Bodies, Policymakers) to Determine Stakeholder Views on the PREM

A two-phase process will be completed–initial data collection followed by a second round of data collection for review and feedback.

##### Initial Data Collection with Service Providers

During data collection for Aim 1, key service staff at each study site will be invited to participate in focus groups to explore how best to ensure meaningfulness, feasibility and to minimise resourcing implications for implementation of a PREM at the service level. Potential participants will also be offered an alternative to do an individual interview if they prefer.

Key clinicians and service managers at participating sites will be identified in collaboration with our partner health services and invited by email or phone. While the exact composition is likely to vary between sites, we anticipate that Aboriginal Health Workers, medical, nursing, and allied health staff, as well as the CQI coordinator and practice manager at each site will be included in the email invitation, with up to 10 staff included at each site. It is anticipated that the majority of invitees will be aware and supportive of the project because of the initial site visits, organisation consent and distributed study information brochures. 

A semi-structured interview guide will be used, with audio recording and verbatim transcription. The focus of the discussions will be on how to ensure the PREM that is developed is useful to the service for meeting accreditation requirements and quality improvement, while being manageable to administer. We will explore their perspectives on any local issues that need to be considered as well as emerging policy requirements. For example, whether the purpose of the PREM is to obtain some structured feedback but also as a permission generating exercise for more detailed conversations/engagement. 

##### Analysis

Similar to the data analysis approach in Aim 1, staff interviews will be audio-recorded and transcribed; and transcripts, memos and field notes will be coded with particular attention paid to variation by remoteness, service size and type. 

##### 2nd Focus Groups with Service Provider Participants 

Following data collection for Aim 2, staff participants from the earlier focus groups/interviews at each site will be invited to participate in a 2nd group process to: (a) review a summary of the analysis from the initial staff focus groups and provide feedback, enhancing rigour; (b) review the framework generated from Aim 1; (c) review the priority ranking by consumers at their site; and (d) discuss actions that could be taken to address the consumer feedback. 

This local site feedback serves two purposes–firstly gaining comment on the relevance of the framework and identified site priorities; and secondly providing services with critical information to use in action planning to improve service delivery.

##### Analysis

Discussions from 2nd focus groups with service provider participants will be audio-recorded, transcribed, coded and analysed thematically. To further enhance the rigour of the study, and support triangulation of findings, these data will be considered with findings from the data from Aims 1 & 2 outlined above. 

#### 2.5.3. Aim 3: Develop a PREM That Captures Valued Experiences of Care in Indigenous PHC Settings; Assess Its Face and Content Validity; Ensure Acceptability to Stakeholders 

Concept prioritisation and factor reduction:

We anticipate identifying a large number of domains and factors, which will need to be prioritised to ensure the PREM is suitably brief. Prioritisation will occur in three stages: (a) consumer prioritisation; (b) service provider review; (c) review against specified criteria for item reduction.

##### Consumer Prioritisation

Consumer prioritisation is critical to ensure the PREM captures elements of quality and experiences of care that are most valued by Indigenous people. We will use a modified Nominal Group Technique, which is well suited to consensus-building among lay people [37]. The Experience of Care Framework will be presented back to the original participants of the first yarning circles as a concept map in a 2nd round of YCs, to enable reflection on the framework and confirmation that it represents the important issues, thus validating the framework and enhancing rigour. Minor revisions and refinements may be made to the framework following this process. Participants in the YCs will discuss and vote for their top five domains, and then factors within domains, using a dot-voting process [38] that generates further discussion and opportunities for revision. 

We will also explore community participants’ views on mode of administration of the PREM, such as use of paper or electronic devices, need for assistance from an Aboriginal Health Worker or aide; and whether the questions need to be translated into site-specific local Indigenous languages. 

It is important to include the initial members of the YCs at each site in this continuation process as they will be able to reflect on the earlier discussions and ensure the concept map reflects these and captures the important issues. If any of the earlier participants choose not to participate in this 2nd YC, this choice will be respected A similar process will be followed as for the initial YCs. Participants will receive a $50 gift card as in Aim 1 to acknowledge their contributions and expertise. Any new community participants participating in the 2nd round of YCs will also receive a VOICE shirt (if not received during the 1st round of YCs).

##### Analysis 

Discussions will be recorded, transcribed and checked for accuracy. The data will be analysed using the same approach as for Aim 1 to finalise the framework, and to understand the process of prioritisation. The domain and factor rankings will be tabulated and compared across the different sites. The domain and factor rankings and the analysis of comments will be reviewed by the Advisory Group, who will decide whether factors should be retained, discarded or combined. Criteria will include ranking by the YCs, whether all domains are represented, whether any factors deemed important by the Advisory Group have been dropped, and commonality or diversity across sites/settings. A reduced list of factors for inclusion will be generated from this analysis. 

##### Service Provider/Stakeholder Review

Clinicians, service managers and policy partners will review the reduced list of factors using an anonymous online survey. The survey will consider the utility of factors against criteria evaluating: (1) amenability to change by the service; (2) likely capacity for service provision to address the factor in improving quality of care; (3) applicability for/to accreditation requirements (to avoid duplication and align various administrative requirements); and (4) unlikely to have strong floor/ceiling effects. It will also collect descriptive information about the participant including role, years at the service, total years in their professional role and Indigenous identity. 

Key clinicians and service managers at participating sites will be identified in collaboration with our partner services and invited by email to participate in the online survey, with a survey link included in the email. Additionally, our policy partners on the project, including RACGP, will identify key staff members to include in the survey. The online survey will be administered using a Research Electronic Data Capture (REDCap) database [39] specifically designed for the study. REDCap is a secure web-based application that can be used for projects to support clinical and translational research.

##### Analysis 

The ratings of utility will be tabulated, and the comments thematically analysed and interpreted, then reviewed by the Advisory Group. 

##### Review against Criteria for Item Reduction

The research team will review findings from the service provider review against pre-specified criteria using a similar process to that used for the Australian Hospital Patient Experience Question Set (AHPEQS) development and consistent with international guidance [40]. The quality criteria will include relevance, appropriateness, utility, and question construction as well as other factors considered important. The research team will review the criteria used in the AHPEQS development [41] and make recommendations to the Advisory Group for modifications and additions. Following input from the Advisory Group, the research team will apply the revised criteria to each of the items. Recommendations will be presented to the Advisory Group who will decide on inclusion, removal, or modification of items for the next phase. 

Question development and cognitive testing: 

The consolidated factors identified in the process above will be converted to questions, then assessed for face and content validity in an iterative process, with appropriate revisions, to ensure that the final questions and instructions are well understood by consumers.

##### Question Development and Review

The factors will be converted to questions for review by the Advisory Group, who will consider the extent to which each question captures the original factor, the language appropriateness, and the response options. Following revisions, these questions and response options will be sent to external experts in survey methodology, who will be provided with an overview of the purpose, information on the process to date, the original factors, and asked to score each question on importance, and comment on whether further revisions are required to wording or response options. These responses will be recorded in an online survey using a REDCap database specifically designed for the study.

Following appropriate revisions this prototype PREM will be assessed in the cognitive testing phase. 

##### Cognitive Testing

Cognitive testing will be undertaken to assess respondents’ comprehension of the questionnaire, the individual questions and the response options [40]. Two rounds will be conducted, with modifications as required. 

Participants in the cognitive testing phase will cover a range of geographical, gender and age categories, be able to speak English, and not have been involved in previous study processes. They will be recruited through three participating services (one each in metropolitan, regional and remote) for one-on-one interviews while attending for care. In each round, 12 patients across age/gender categories will be recruited at each clinic (total 36 participants/round). While this number is greater than usually used for cognitive testing, it is important to ensure representation of the diverse target population [40] and varying comfort with use of written English in our target population. 

In each interview, participants will be asked to complete the questionnaire on a tablet (e.g., iPad) device. They will be asked to use the ‘think aloud’ approach [40,42], in which participants verbalise their thoughts as they answer questions. If needed, the interviewer will read questions to participants. The interviewer will note time taken, hesitations, body language and indications of difficulty. This step will be followed by discussion to assess whether the item is understood, the perceived relevance, and any difficulties. Interviews will be audio-recorded, transcribed verbatim and summarised. The interviews will take approximately one hour, and participants will receive a $40 gift card in acknowledgement of their contribution.

Following each interview round, the research team will review the findings and make recommendations regarding changes as appropriate, with clear documentation of changes and the rationale. The Advisory Group will review these recommendations and decide on questions to include in the version for Aim 4 psychometric testing (PREM_v1).

#### 2.5.4. Aim 4: Validate the PREM by Assessing Its Psychometric Properties

##### Psychometric Testing

The PREM will be validated by administering PREM_v1 at the original eight sites, with participants recruited while attending for care. The sample size will be determined by the number of items in the PREM, the number of contextual groups we wish to assess for invariance, and a sufficient ratio of participants to variables to support rigorous psychometric testing [43]. A total sample of about 400 completed surveys across the eight sites will likely be required. 

Following their care provision, service clients will be asked to complete the PREM. The PREM will be administered using tablet devices, with facilitator support as required. Participants will be thanked by entry into a prize draw for a fresh food box or a pantry hamper of the partner heath service’s choice at each site.

##### Analysis 

The psychometric properties and scoring system of the PREM will be assessed consistent with the Consensus-based Standards for the selection of health status Measurement Instruments (COSMIN) checklist [44]. The COSMIN checklist contains twelve criteria of which nine contain standards for the included measurement properties focusing on internal consistency, reliability, measurement error, content validity, structural validity, hypotheses testing, cross-cultural validity, criterion validity and responsiveness. The remainder of the criteria contain standards on interpretability, general requirements for application of the Item Response Theory (IRT), and generalisability of the results.

Initial descriptive analysis will include examination of missing data, ceiling and floor effects, and sample characteristics. A classic test theory approach will be undertaken using exploratory factor analysis (EFA) and confirmatory factor analysis (CFA) to determine and then validate the scale structure. Multigroup confirmatory factor analysis (MGCFA) will be used to determine the consistency of structure, item meaning, and operationalisation between groups based on contextual differences, geographic locations or demographic characteristics if sample size permits [45]. Factorability will be assessed using the Kaiser-Meyer-Olkin value [43]. Item reduction will be based on principal factor analysis (PFA) assessments of internal consistency. Items will be removed if they are redundant (Cronbach’s α > 0.8) or do not adequately load on to a factor (<0.3 in PFA). Composite reliability will be calculated to determine convergent validity. Goodness-of-fit will be assessed using Chi-square test, Comparative Fit Index (CFI), Tucker–Lewis Index, and root mean square error of approximation (RMSEA) [46]. MGCFA will use the sequential constraint imposition procedure using a series of nested and progressively more constrained models. 

##### Review and Interpretation 

Results from the statistical analysis will be iteratively discussed and interpreted by the Advisory Group allowing contextualisation of the findings and decisions regarding the final PREM. 

#### 2.5.5. Providing Feedback to Services

Consistent with the action research approach there are multiple points within the project when information will be provided back to participating sites, with discussion regarding the implications and the opportunity for service improvement (Figure 1). These include presentation of initial findings to both the community and the services as part of Aims 2 and 3, and again at the end, when findings for each site, together with summary information for other sites, will be discussed with each service, enabling them to consider implications for their service. 

Two representatives from each service will be invited to attend a ‘service learning’ workshop with other sites, the research team and the Advisory Group at two points to review findings, share learnings and consider implications and actions (see Figure 1). This, together with local site feedback, will ensure practical value and further opportunity for services to be involved as part of the PREM development process.

## 3. Results

The VOICE project is currently in the initial phase that includes setting up the governance structure and processes, setting up sites and conducting initial site visits, and establishing monitoring and reporting processes and tools. Results deriving from this research project will be published at a later stage. 

## 4. Discussion

The VOICE project grew out of discussions between Aboriginal Community Controlled Health Services, Indigenous and non-Indigenous researchers, and policy-level stakeholders in Australia over the past 10 years. These groups identified a need to develop a validated PREM to capture Indigenous Australians’ experiences of care. 

In Australia, there have been some recent advances in development of PREMs for Indigenous people in the areas of cancer care and cultural safety in hospitals [47,48]. This work emphasised the importance of cultural security, trust, relationships, family, and effective communication [47,48]. The NSW Bureau of Health Information has also recently developed an Indigenous-specific PREM for use with hospital patients, focusing on the processes of admission, stay and discharge, again emphasising family involvement, respectful care and communication [49]. The Southcentral Foundation in Anchorage, Alaska-a high-performing PHC service for Native Alaskans-has developed a culturally appropriate patient satisfaction scale [50]. While patient satisfaction differs from experience, there are valuable lessons from this project as it has led to measurable improvements in quality of care. This iPad administered scale is completed following a clinic visit and, for those with limited literacy, is facilitated by an Alaskan native customer service officer. Results are tracked in real time enabling rapid response. Consumer evaluations are used internally to reorient service delivery to better serve consumers, using rapid-cycle quality improvement methods. Southcentral Foundation have demonstrated improvements in service delivery, customer satisfaction and strengthened relationships with their consumers [50]. Despite these developments, there are currently no PREMs for use in PHC services in Australia that have been developed and validated with Indigenous Australians–the VOICE project aims to address this gap.

At all levels within the research team and the study itself, Indigenous Australians will lead, be consulted and benefit from this project. This study, from its aims to data collection and benefits to the community, aims to be a case study in excellent community engagement with Indigenous Australians.

The project is being co-led and implemented by the Indigenous and non-Indigenous project team members, in collaboration with PHC services and other stakeholders ensuring co-design of a tool that is fit for purpose for the communities they serve. The shared knowledge gained through community engagement, development of the Experience of Care Framework, the local site feedback, the ‘service learning’ workshops, and the newly developed or consolidated relationships will enable participating services and communities to both contribute to, and benefit from the research. This process has potential to drive improvements in the quality of healthcare provided to Indigenous Australians, enhancing cultural security, consumer experiences, and engagement with care, contributing to improvements in health outcomes.

We envisage undertaking a subsequent study to explore opportunities for routine implementation of the PREM to support continuous quality improvement in addition to accreditation. This study would identify resourcing requirements, challenges and factors that would enable widescale adoption of the instrument.

## 5. Conclusions

The final PREM and resources for its use will be developed by the VOICE project, considering inputs from key stakeholders and the Advisory Group at all stages of the research. Our final product, a validated Indigenous-specific PREM, will be supported by the RACGP for accreditation of PHC services, and will be made freely available, thus supporting translation into policy and practice. Critically, service and community input in the co-design process will ensure it meets service needs for CQI processes and reflects the priorities and values of Indigenous people. It is envisaged that use of the PREM in CQI processes will increase culturally secure and positive care experiences, enhancing consumer engagement, and contributing to improved health outcomes.

## Data Availability

Not applicable.
